# Finite element analysis of titanium anatomic plate and titanium reconstructive plate for treatment of extra-articular fractures of the scapula

**DOI:** 10.1186/s13018-023-03614-x

**Published:** 2023-02-23

**Authors:** Yanliang Shang, Yunlong Bi, Yang Cao, Yansong Wang

**Affiliations:** Department of Orthopedic Trauma, First Affiliated Hospital of Jinzhou Medical University, Jinzhou, China

**Keywords:** Scapula fracture, Finite element analysis, Anatomic plate, Reconstructive plate

## Abstract

**Background:**

Due to the lack of postoperative reporting outcomes and bio-mechanical studies, an optimal management of scapular fractures has not been well-established in clinical treatment, even though there are many options available. This study aimed to compare the stability of the new titanium anatomic and traditional titanium reconstructive plates for extra-articular scapular fractures through finite element analysis.

**Methods:**

Two models of scapular assembly were constructed, including one anatomic plate (AP model) and one reconstructive plate (RP model). After meshing, material parameter, and boundary condition settings, we applied four loading conditions to simulate forces acting on the scapula and osteosynthesis material. To evaluate the bio-mechanical properties, the equivalent von Mises stress, equivalent elastic strain, and total deformation were investigated.

**Result:**

The stress and strain distribution of model AP has better performance than model RP, with more uniform and lower values. The maximum stress value of the scapula in model AP is smaller than that of the scapula in model RP (102.83 MPa vs. 166.71 MPa). The maximum stress of the anatomic plate is half that of the reconstructive plate (218.34 MPa vs. 416.01 MPa). The maximum strain of the scapula in model AP is smaller than that of the scapula in model RP (0.0071 vs. 0.0106). The maximum strain of the anatomic plate is half that of the reconstructive plate (0.0019 vs. 0.0037). The maximum displacement of each model is all at the acromion, with a similar value (2.2947 mm vs. 1.8308 mm).

**Conclusions:**

With sufficient bio-mechanical stability, the anatomic plate to support scapular fracture fragments was superior to that of the reconstructive plate.

## Background

Scapular fractures are often caused by high-energy injuries, accounting for about 1% of all body fractures [1]. In recent years, the number of patients with scapular fractures has increased due to frequent traffic accidents. Surgical treatment of unstable scapular fractures can reduce postoperative chronic pain, improve shoulder joint function, and facilitate early recovery, which has gradually become the common consent of orthopedic surgeons [2, 3].

Due to the special structure of the scapula and the different types of fractures, there is no standard internal fixation device available for scapular fractures in clinical treatment. Although there are many internal fixation devices available, such as the calcaneal plate, reconstructive plate, T-type plate, Y-type plate, micro-plate, etc.[4–6].

Among them, the reconstructive plate is the most widely used [7]. The reconstructive plate usually needs to be pre-bent, which is inconvenient and prolongs the operation time. In addition, pre-bending has a certain impact on the stability of the reconstructive plate, thereby increasing the risk of complications such as secondary fractures and malunions [8].

Considering these problems and the reasons for the risk of complications, a novel titanium anatomic plate was designed for scapular fractures. Zhang et al. retrospectively analyzed the clinical data of 41 scapular fracture patients with at least 12 months of follow-up [9]. Compared with the reconstructive plates, the anatomic plates had obvious advantages that could shorten the operation time, reduce intraoperative blood loss, and promote the early recovery of patients.

To further evaluate the supporting effect and bio-mechanical properties of the new titanium anatomic plate on scapular fracture fragments, we used finite element analysis to compare anatomic and reconstructive plates for scapular extra-articular fractures from the perspective of mechanics.

## Materials and methods

### Patient history and scapular model construction

A 35-year-old male volunteer (72.0 kg and 1.76 m) with no history of scapular injury or variant participated in the study. The written consent of the volunteer was obtained.

**Fig. 1:**
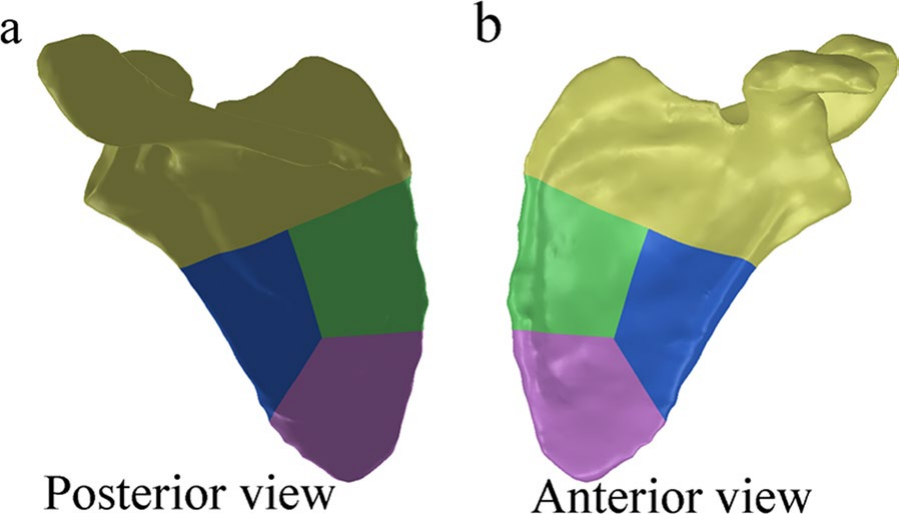
Scapula fracture model

First, high-resolution computed tomography (CT) scans of the scapula were performed on a dual-source 64-slice CT scanner (Sensation64, Siemens, Germany) with a tube current of 259 mA and a tube voltage of 120 kV. Slice thickness and spacing were 0.5 mm. Digital Imaging and Communications in Medicine (DICOM) images with 512 by 512 pixels were obtained by scanning layer by layer from top to bottom. Then, the CT data were imported into Mimics 19.0 (Materialise, Leuven, Belgium) to preliminary reconstruct the scapular model. Finally, the scapular model was imported into SolidWorks 2010 (Dassault Systemes, Massachusetts, USA), the fracture line was cut, and the scapular fracture model was established after smoothing (Fig. 1).

### Building the two internal fixation models of extra-articular scapular fractures

**Fig. 2:**
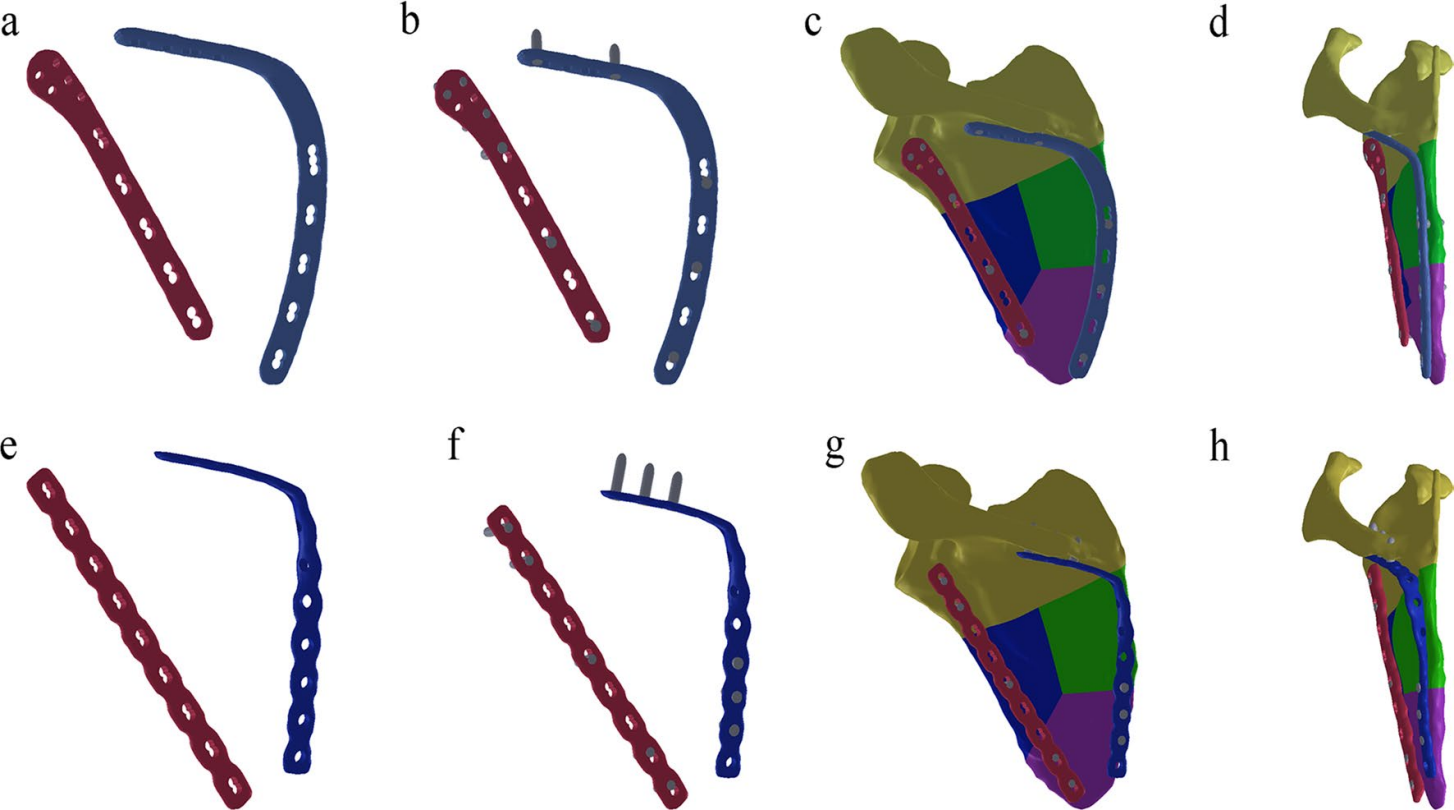
Internal fixation devices were fabricated and then placed on the back of the scapular models: **a**–**d** AP model, **e**–**h** RP model

Titanium plates and locking screws were fabricated using Mimics 19.0 and SolidWorks 2010 in the same way. Two types of titanium plates and screws (IRENE, Tianjin, China) were scanned by CT, and the tomographic data were imported into Mimics 19.0 to construct the models. The obtained models were imported into SolidWorks 2010 for smoothing to obtain the 3D solid models. Because the length and diameter of the screws were shorter, all screws were simplified into 3.5-mm and 5.2-mm solid columns. According to the actual size of the steel plate hole, 2–3 screws were placed at the distal and proximal ends of the plates. The two different plates with screws were placed on the back of the scapular models, respectively (Fig. 2).

### Meshing and material properties

**Fig. 3:**
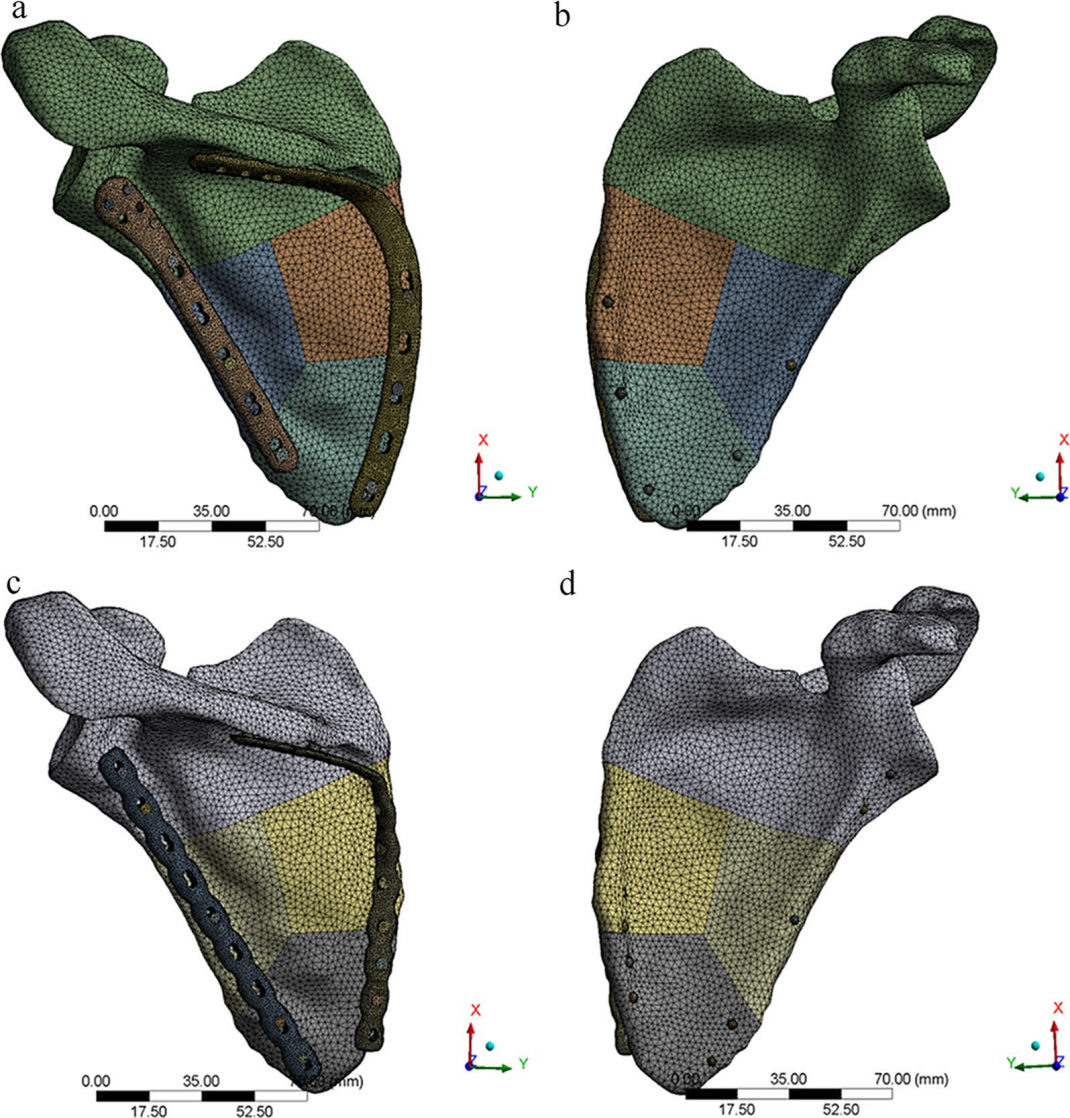
Model after meshing: **a** posterior view of AP model, **b** anterior view of AP model, **c** posterior view of RP model, **d** anterior view of RP model

The models were imported into ANSYS Workbench 20.0 (Swanson, Houston, USA). Two models were meshed as shown in Table 1 and Fig. 3. The element type was Solid187. The titanium alloy Ti6Al4V was used to model the implant material. The bone was defined with linear elastic material properties. According to the gray scale of CT, cortical bone and cancellous bone were distinguished so as to set different material parameters. The elastic modulus and Poisson’s ratio of various structural materials are shown in Table 2 [10–12].

**Table 1:** Nodes and elements of two kinds of models

Model	Nodes	Element number
AP model	248,329	154,281
RP model	226,976	140,639

**Table 2:** Material properties of models

Materials	Elastic modulus (MPa)	Poisson ratio
Cortical bone	17,000	0.30
Cancellous bone	1600	0.30
Plate	114,000	0.30
Screw	114,000	0.30

### Boundary conditions and loading force settings

**Fig. 4:**
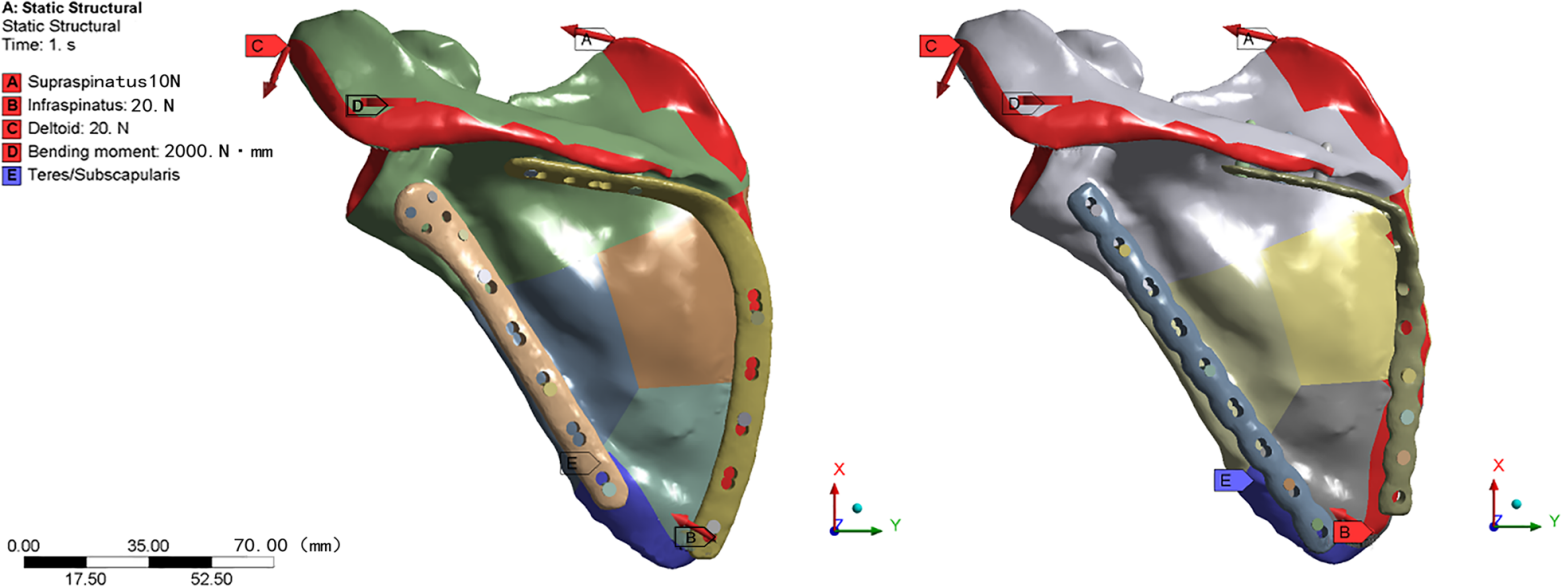
Loading and boundary conditions: **A** supraspinatus (10 N), **B** infraspinatus (20 N), **C** deltoids (20 N), **D** bending moment (2000 N mm), **E** teres minor, teres major, and subscapularis muscles (Fixed restraints)

The screw-plate and screw-bone interfaces were all bonded. The contact interface of fracture fragments and plate-bone were defined as frictional, with a friction coefficient of 0.37 [13, 14]. As shown in Fig. 4, the three major muscle forces of the supranational (10 N), infraspinatus (20 N), and deltoids (20 N) were simulated to describe the load on the scapula during extension. In order to further simulate the torque of abduction action on the scapula, an outward bending moment of 2000 N mm was applied along the axis of the scapula, with the glenoid of the scapula as the stress point. Fixed restraints were applied to the part of the scapula that was acted on by the teres minor, teres major, and subscapularis muscles [15, 16].

## Results

**Fig. 5:**
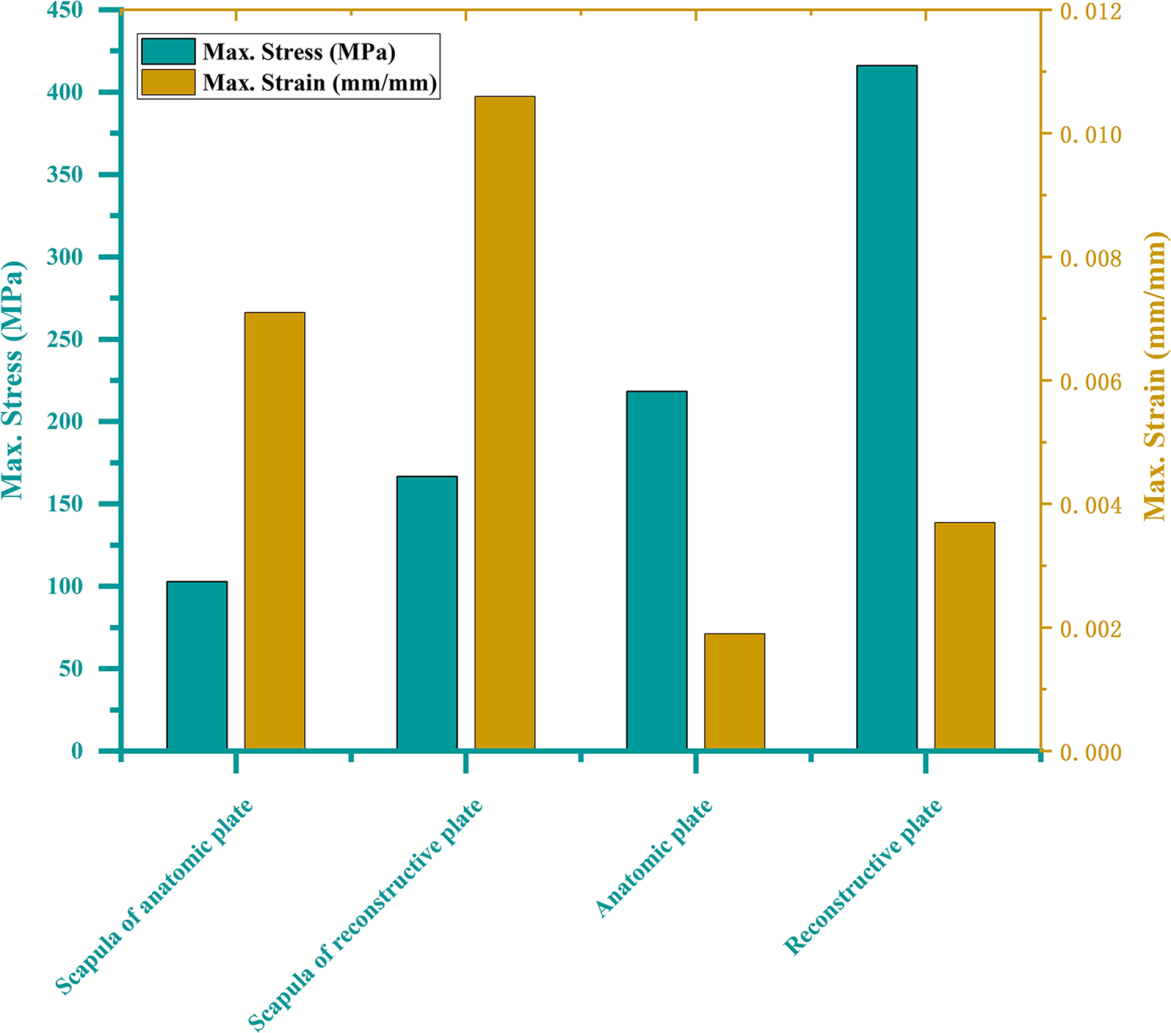
Peak stress and peak strain of different models

**Fig. 6:**
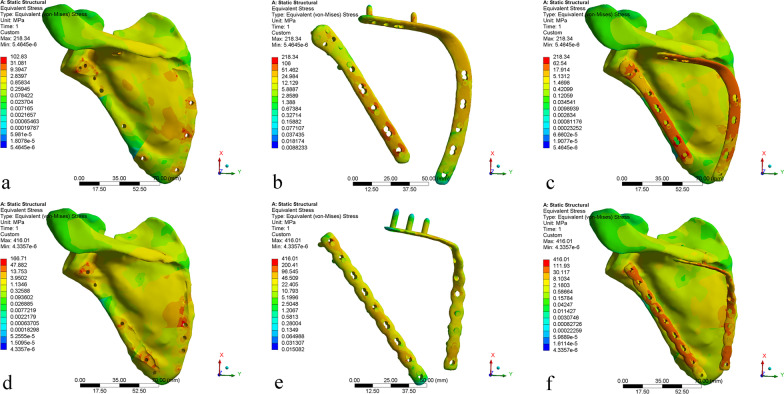
von Mises stress distribution: **a**–**c** AP model, **d**–**f** RP Models

**Fig. 7:**
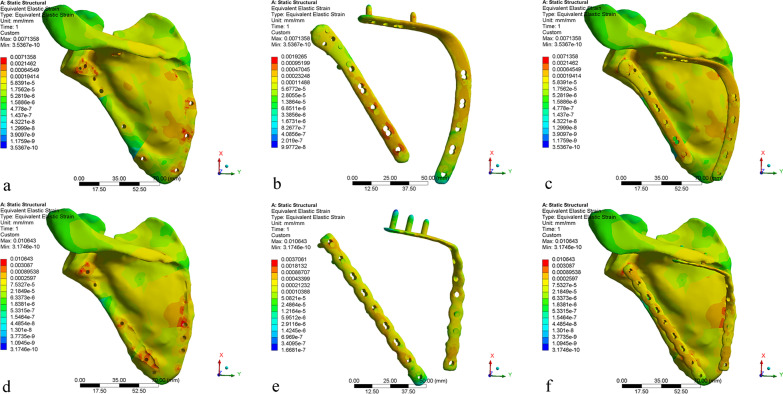
Equivalent elastic strain distribution: **a**–**c** AP model, **d**–**f** RP Models

**Fig. 8:**
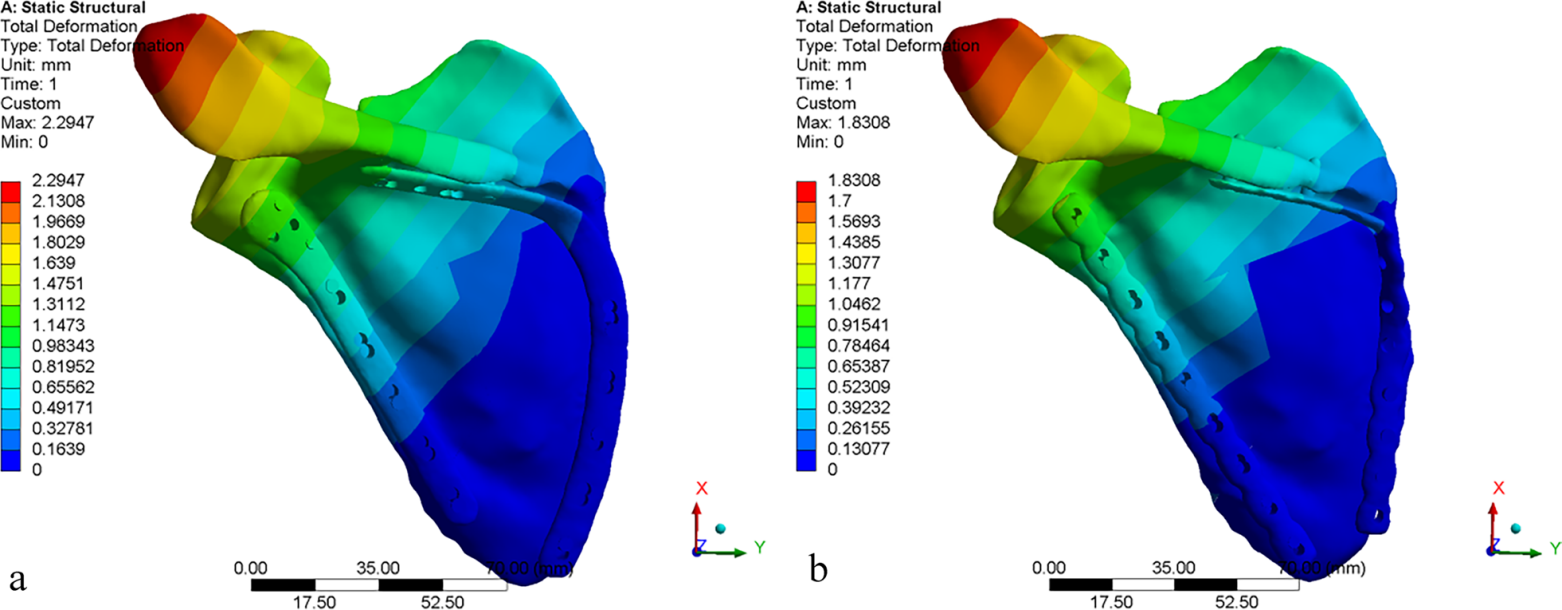
Total deformation: **a** AP model, **b** RP Models

The von Mises stress, equivalent elastic strain, and total deformation are studied to gain a better understanding of the results. The results are discussed in three sub-sections in this article. For the results of von Mises stress and equivalent elastic strain, with more concentrated and higher values indicating worse performance [17], a comparative summary of the stress and strain among the reconstructed scapula and different internal fixation devices is presented in Table 3 and Fig. 5. In addition, the results of stress, strain, and displacement distribution are obtained within the same color bar range for different models, as shown in Figs. 6, 7, and 8. This allows them to compare the results immediately in the rainbow colors, which represent the distribution.

**Table 3:** Maximum and average values of stress and strain

	Scapula of anatomic plate	Scapula of reconstructive plate	Anatomic plate	Reconstructive plate
Stress (MPa)				
Max	102.83	166.71	218.34	416.01
Avg	0.95	1.43	17.21	22.42
Min	5.4645^−^^6^	4.3357^−^^6^	0.008823	0.015082
Strain (mm/mm)				
Max	0.0071	0.0106	0.0019	0.0037
Avg	0.00006590	0.00010133	0.00014908	0.00019456
Min	3.5367^−^^10^	3.1746^−^^10^	9.9772^−^^8^	1.6681^−^^7^

### Equivalent von Mises stress

As shown in Fig. 6, the stress distribution of the scapula is uneven. There are obvious stress concentrations near the fracture lines of both models. In addition, the medial border and scapula neck of both models have relatively concentrated areas of stress. The maximum stress of the scapula in model AP is smaller than that of the scapula in model RP, with values of 102.83 MPa and 166.71 MPa, respectively. After the plates and screws are fixed, a certain stress concentration phenomenon appeared, concentrating mainly on the internal fixation device. The maximum stress of the anatomic plate is half that of the reconstructive plates (218.34 MPa vs. 416.01 MPa). In the anatomic plate, the maximum stress occurs at the edge of the screw hole at the bottom of the lateral border row. In the reconstructive plate, the maximum stress occurs at the edge of the first screw hole at the top of the lateral border row.

### Equivalent elastic strain

As shown in Fig. 7, the strain distribution of the scapula in model AP is mainly concentrated on the medial border and scapula neck. The strain distribution of the scapula in model RP is distributed along the medial and lateral borders. Meanwhile, the maximum equivalent elastic strain of the scapula in model AP is smaller than that of the scapula in model RP, with the values of 0.0071 and 0.0106, respectively. The maximum strain of the anatomic plates is half that of the reconstructive plates (0.0019 vs. 0.0037). In the anatomic plate, the maximum strain occurs at the edge of the screw hole at the bottom of the lateral border row. In the reconstructive plate, the maximum strain occurs at the edge of the first screw hole at the top of the lateral border row.

### Total deformation

As shown in Fig. 8, the maximum displacement in each group is at the acromion. This may be because of the traction effect of the deltoids muscle, which simulates the force characteristics after surgery. Meanwhile, the maximum displacement of the scapula body is less than 2 mm in each model, which indicated that the two different internal fixation devices are reliable [18]. The maximum displacements of models AP and RP are similar (2.2947 vs. 1.8308), with a difference of only 0.4 mm.

## Discussion

Conservative treatment of scapular fractures will cause multiple complications, such as residual pain, impingement, and scapular dyskinesia. Regardless of the treatment methods, the purpose of treatment is to restore the function of the scapula and prevent the occurrence of complications. For unstable extra-articular scapular fractures, timely surgical treatment can restore the stability of the scapula and promote early recovery of function [2, 3, 19–21].

Due to the irregular shape of the scapula, each part has its own unique anatomy. During the surgery, different internal fixation devices should be selected according to the type of scapular fracture [4–6]. The reconstructive plate was one of the most commonly used internal fixation devices in scapular fractures [7]. Before the reconstructive plate was fixed, it needed to be pre-bent several times so that it could be as close to the fracture surface as possible. However, repeated pre-bending would result in fatigue fractures of the internal fixing, which could affect the stability of the material [8].

In view of the deficiencies of traditional internal fixation devices, a new titanium anatomic plate was designed based on the 3D printed models of the scapula of 62 patients and the anatomical data of related literature [22–24]. The titanium anatomic plate was a double-row internal fixation device that followed the design principle of “three-point, two-line” [25]. One row of anatomic plates runs from the backside of the spine scapula to the inferior angle along the medial border. Another row of plates runs from the neck of scapula to the inferior angle along the lateral border. In order to avoid iatrogenic glenoid injury, the proximal screw guide hole of the anatomic plate was designed with an inward designed. The scapula neck was designed as an interlocking system of five screw holes with different angles. Meanwhile, the anatomic plate was placed on the thickened part of the lateral border, which effectively increased the fixation strength and avoided screw loss [26]. Design the edge of the anatomic plate as a low-notch structure to reduce soft tissue irritation. The basic structure of the anatomic plate fits with the overall shape of the scapula without further bending. For patients with complex scapular fractures, it is not necessary to prepare multiple internal fixation instruments before surgery. The anatomic plate can simultaneously fix the scapular neck, inner edge, lateral edge, and scapular ridge. We chose titanium alloys, which are widely used commercially, considering the availability and accessibility of customization scapula anatomic plate materials. Some novel materials have been reported, such as fiber-reinforced composites, which can alleviate the stress shielding effect with outstanding mechanical properties [27].

Zhang et al. previous study showed that the anatomic plate could avoid excessive pre-bending and reduce operative time and blood loss during the intraoperative [9]. However, this was a retrospective study limited by the number of clinical cases. Therefore, we need to further evaluate titanium anatomic plates in a new way.

In recent years, with the development of computer image processing technology, finite element analysis has been applied to various fields, which has greatly promoted the development of bio-medicine [28]. The emergence of finite element analysis provides a new idea for the study of biomechanics. In our study, we used finite element analysis to comprehensively evaluate the new scapular anatomic plate from the perspective of mechanics.

The model AP provides more stable fixation than model RP. As shown in Fig. 6, the stress concentration of the scapula at the fracture lines is reduced after installing the internal fixation devices. The internal fixation material can share a part of the stress concentration, so that the stress concentration on the fracture lines is reduced. The stress distribution around the fracture lines reflects the influence of local stress on fracture healing to a certain extent [17]. The stress distribution of anatomic plates has better performance, with more uniform and lower values. In contrast, the reconstructive plate is more prone to micro-fracture under the action of shear force, resulting in internal fixation fatigue and even screw fracture. The yield strength of titanium alloy is usually 825 MPa, although different suppliers are slightly different. The maximum stress of each component is less than the yield strength of titanium alloy, indicating that the titanium alloy can be trusted as a scapular reconstruction material [29, 30]. In addition, calculate the security factor of the two steel plates (security factor = yield strength/maximum stress). The safety factor of the anatomic plate is 825/218.34 = 3.7785; the safety factor of the reconstructive plate is 825/416.01 = 1.9831. It can be found that the anatomic plate safety factor is higher. As shown in Fig. 7, the anatomic plate is superior to the reconstructive plate in terms of strain distribution. The strain distribution of the lateral border row in model AP is more dispersed, which may be because of the interlocking system of five screw holes with different angles designed near the scapula neck. Meanwhile, due to the low-notch design of the edge, the strain distribution of the overall anatomic plate is more uniform, which also indicates that our design conforms to the anatomical specifics of the scapula. As shown in Fig. 8, the total deformation of the two different models is similar. Overall, the design of the anatomic plate is more suitable for the scapular structure, providing a good mechanical environment with a stable and lasting holding force. These are conducive to reducing the risk of complications such as secondary fractures and malunions to some extent.

The limitations of our study must be considered. One limitation of this study is that because the length and diameter of the screw are short, all screws were simplified into solid columns. Therefore, the details of screws such as hollow and thread were not considered. Furthermore, the impact of the width of the fracture gap on the results was not considered in the scapular model. The fracture gaps in this simplified scapular model were flat and fit each other. But the actual fracture line usually has a certain gap, and the edges are not aligned. In this case, it often produces greater friction than the simplified scapular model, leading to centralized stress. But this is not the main purpose of this study. Therefore, the above optimization of the scapular model was made. The third limitation of this study is that the four loads we simulated were under ideal conditions and might differ from actual motion. This is a common obstacle to the current finite element analysis studies. Further experiments with internal fixation device models and scapular models should be performed to determine the influence of different loads and fixed boundary conditions at the muscle attachment points of each model.

To the best of our knowledge, this is the first study on the bio-mechanical stability of the new titanium anatomic plate in the treatment of scapular extra-articular fractures. We combined this with the finite element analysis to comprehensively evaluate the new scapular anatomic plate from the perspective of mechanics, which provides a theoretical basis for the clinical application.

## Conclusions

With sufficient bio-mechanical stability, the anatomic plate to support scapular fracture fragments was superior to that of the reconstructive plate. The study provides a good theoretical basis for subsequent clinical investigations of the various scapular therapeutic procedures in the future. It is recommended to fix the scapular fracture with the titanium anatomic plate.

## Data Availability

Not applicable.

## Abbreviations

AP model: Anatomic plate model
RP model: Reconstructive plate model
CT: Computed tomography
DICOM: Digital Imaging and Communications in Medicine
